# Ligand selectivity in tachykinin and natalisin neuropeptidergic systems of the honey bee parasitic mite *Varroa destructor*

**DOI:** 10.1038/srep19547

**Published:** 2016-01-28

**Authors:** Hongbo Jiang, Donghun Kim, Sharon Dobesh, Jay D. Evans, Ronald J. Nachman, Krzysztof Kaczmarek, Janusz Zabrocki, Yoonseong Park

**Affiliations:** 1 Department of Entomology, Kansas State University, Manhattan, Kansas 66506, United States; 2 Key Laboratory of Entomology and Pest Control Engineering, College of Plant Protection, Southwest University, Chongqing 400715, People’s Republic of China; 3 Bee Research Laboratory, BARC-E, USDA-Agricultural Research Service, Beltsville, MD 20705, USA; 4 Insect Control and Cotton Disease Research Unit, Southern Plains Agricultural Research Center, USDA, 2881 F/B Road, College Station, TX 77845, United States; 5 Institute of Organic Chemistry, Lodz University of Technology, 90-924 Lodz, Poland

## Abstract

The varroa mite, *Varroa destructor*, is a devastating ectoparasite of the honey bees *Apis mellifera* and *A. cerana*. Control of these mites in beehives is a challenge in part due to the lack of toxic agents that are specific to mites and not to the host honey bee. In searching for a specific toxic target of varroa mites, we investigated two closely related neuropeptidergic systems, tachykinin-related peptide (TRP) and natalisin (NTL), and their respective receptors. Honey bees lack both NTL and the NTL receptor in their genome sequences, providing the rationale for investigating these receptors to understand their specificities to various ligands. We characterized the receptors for NTL and TRP of *V. destructor* (VdNTL-R and VdTRP-R, respectively) and for TRP of *A. mellifera* (AmTRP-R) in a heterologous reporter assay system to determine the activities of various ligands including TRP/NTL peptides and peptidomimetics. Although we found that AmTRP-R is highly promiscuous, activated by various ligands including two VdNTL peptides when a total of 36 ligands were tested, we serendipitously found that peptides carrying the C-terminal motif -FWxxRamide are highly specific to VdTRP-R. This motif can serve as a seed sequence for designing a VdTRP-R-specific agonist.

Alarming population declines of honey bees in recent years are at least partly due to the ectoparasitic honey bee mite, *Varroa destructor*^1^,^2^. This mite is considered to be the major threat to apiculture, not only for its direct damage to the colony, but also for being a vector of several important bee viruses^3^,^4^,^5^. Thus, the need to develop novel control methods against the varroa mite is urgent. The development of selective acaricidal methods is based on understanding the biological, physiological, and toxicological differences between the parasitic mite and the insect host.

A disruption of a neuropeptidergic system by using peptidomimetics specifically appeals in this case^6^,^7^. Small peptides including unnatural amino acids can be designed to improve the specificity and bioavailability of the compounds for the target peptide receptor^8^,^9^. Furthermore, a major limitation to the practical use of peptidomimetics in the field, the high costs for large-scale synthesis, can be overcome by the application of peptidomimetics at a small scale only in the beehives.

We have attempted to discover differences in the neuropeptidergic systems between the honey bee and varroa mite. Comparing the list of neuropeptides between the initial draft of the genome sequence of the varroa mite and the honey bee genome sequence, we found that the honey bee lacks a neuropeptide natalisin (NTL), while the varroa mite retained the NTL peptide with the consensus motif. Both species retain a closely related neuropeptide tachykinin-related peptide (TRP). This difference motivated us to examine whether the NTL signaling system, which is lacking in the honey bee, can serve as a specific acaricidal target in apiculture.

NTL and TRP are closely related neuropeptides with the common sequence motif FxxxRamide (C-terminally amidated). The most common insect TRP consensus C-terminal sequence is FxGxRamide, while the NTL consensus sequence varies in an Order-specific manner: FxPxRamide for Diptera and FWxxRamide for Lepidoptera and Hemiptera^10^. Likewise, the receptors for each NTL and TRP are closely related each other, but unequivocally form separate clusters. NTL is involved in the reproduction of *Drosophila melanogaster* and *Tribolium castaneum*^10^. TRP is a multifunctional peptide found in various insects^11^. Its functions include myotropic activity^12^, modulation of olfactory neurons^13^,^14^, male aggressive behavior^15^, diuretic function in the Malpighian tubules^16^, and control of lipid metabolism^17^. In agreement with the significant biological functions reported for the NTL/TRP signaling system, RNA interference ubiquitously suppressing the TRP in *D. melanogaster* resulted in embryonic lethality^14^. Importantly, biostable TRP peptidomimetics were shown to have potent aphidicidal activities by feeding^18^.

In the current study, we cloned the NTL and TRP receptors in the varroa mite and the TRP receptor in the honey bee. We examined the ligand specificities of these receptors in a heterologous reporter system with endogenous ligands and peptidomimetics. The data demonstrating a difference in ligand specificities of the receptors provide a foundation for the development of novel varroa-mite-specific control agents.

## Results and Discussion

### NTL and *TRP in V*. destructor and *A. mellifera*

**Figure 1:**
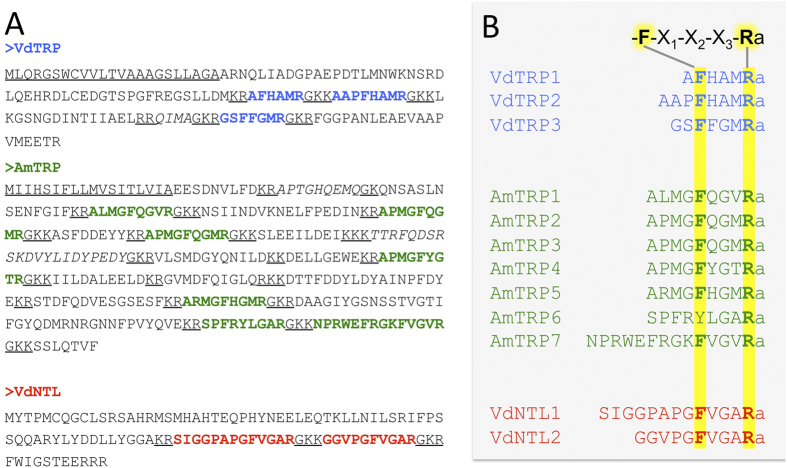
The VdNTL, VdTRP, and AmTRP peptides show unique C-terminal motifs. (**A**) Deduced amino acid sequences for the predicted precursors of the NTL and TRP peptides from *V. destructor* and *A. mellifera*; (**B**) Alignment of putative mature peptides showing the C-terminal motifs with the variations. The highly conserved residues F and R are highlighted.

**Figure 2:**
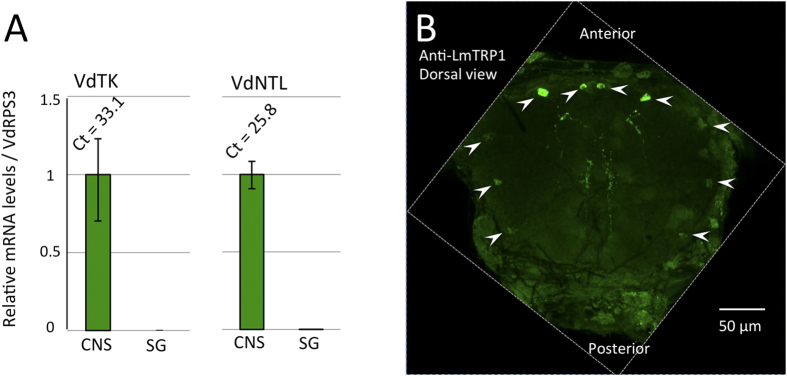
Transcript levels for each VdTRP and VdNTL in the central nervous system (CNS, synganglion) and in the salivary gland (SG). (**A**) RT-PCR testing the presence of both *ntl* and *trp* transcripts in two different tissues, the CNS and SG, of the phoretic varroa mite. The values are normalized by RPS3 transcript level; (**B**) Dorsal view of the immunoreactivity of the *V. destructor* synganglion to the antibody (LmTRP1). Positive neuronal cell bodies are indicated by arrowheads. Scale bar = 50 μm.

Both TRP and NTL in other arthropods are characterized by the precursors containing multiple mature peptides with dibasic-, or monobasic-cleavage sites (Fig. 1). The predicted TRP precursor in *V. destructor* consists of 147 amino acids and has three putative mature peptides sharing the C-terminal motif; these are known to conform to the typical insect TRP motif with M (Met) in the X3 position (FXXMRamide) (Fig. 1). In the honey bee, the TRP contains six putative mature peptides counting only the peptides carrying the FxxxRamide motifs. Although the mature peptides are more varied than those in the varroa mite, three of them still have the typical insect TRP motif FXGMRamide. The G (Gly) in the X2 position is a common feature of all six peptides. In addition, there are two associated peptides (AP) carrying the C-terminal amidation motif in the honey bee *trp* gene, but without the typical TRP consensus: AmTRP-AP1, APTGHQEMQamide and AmTRP-AP2, TTRFQDSRSKDVYLIDYPEDYamide (Fig. 1). The predicted NTL precursor in *V. destructor* appears to be incomplete in the 5′ end for the sequence encoding the signal peptide. The incomplete sequence is composed of 105 amino acids (Fig. 1), including 2 putative mature peptides sharing an identical C-terminal heptapeptide (PGFVGARamide). Although the 5′ end of the open reading frame may be incomplete, presence of additional mature peptide in the precursor is unlikely. The most closely related sequence NTL from *Metaseiulus occidentalis* also contains only two predicted mature peptides. In addition, we were unable to identify any similar motifs in the genome sequence, although the completeness of the genome remains as a question yet. Both of the mature peptides contain the predicted amidation sequence motifs at the C-terminus, which are the same as those of the NTL and TRP peptides in *D. melanogaster, T. castaneum* and *B. mori*^10^. There were no amino acid residues that clearly discriminated VdNTLs from AmTRPs by their FXGXRamide C-terminal motifs; the N-terminal amino acid residues may provide the functional differences that distinguish the different receptors. We tested the expression patterns of VdNTL and VdTRP in the salivary glands (SG) and the central nervous system (CNS) because we suspected a possible role of VdNTL as a salivary protein of the ectoparasite that affects the host system. Sialokinin of a mosquito species *Aedes aegypti* and Eleidosin in *Octopus*, both tachykinin-related peptides, are known to be the salivary proteins utilized for attacking their host or prey^10^. This postulate was also based on the high similarity of the C-terminal motif of VdNTL to that of AmTRP and its strong cross-activity with the AmTRP-R (see next section). However, the results of reverse transcription-PCR for the SG and CNS samples from phoretic mites showed that both the VdNTL and the VdTRP transcripts were relatively abundant in the CNS, but undetectable in the SG (threshold cycle Ct >35, Fig. 2A). This hypothesis needs to be further tested in the mites in bee brood cells. Immunohistochemistry using the antibody raised against *Locusta migratoria* TRP1 (LmTRP1 as GPSGFYGVRamide)^19^ showed immunoreactive cells in the CNS; two pairs of protocerebral neurons with posterior projections and three pairs in segmental pedal neurons. Other antibodies raised against NTLs of *D. melanogaster*, DmNTL4 and DmNTL5 (HRNLFQVDDPFFATRa and LQLRDLYNADDPFVPNRa, respectively) did not show immunoreactive cells in the varroa mite CNS. The positive immunoreactivity for the anti-LmTRP1 antibody could be for VdTRPs or VdNTLs, or both based on the moderate levels of similarities to the C-terminal motif of the immunogen, or for unknown cross reactivity.

### Receptors for NTL and TRP in *V. destructor* and *A. mellifera*

**Figure 3:**
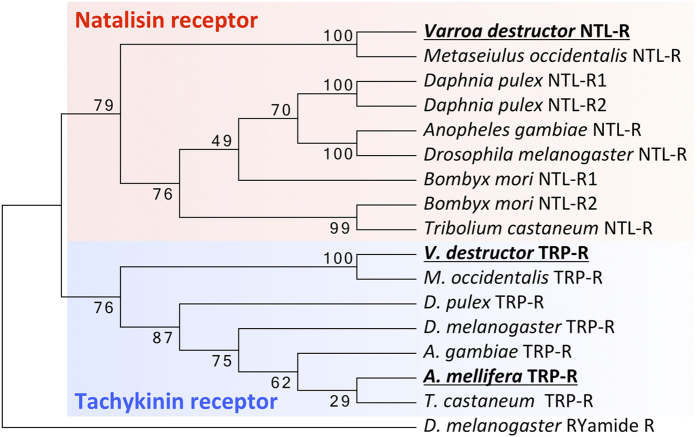
Two separate clades for NTL-R and TRP-R, containing VdNTL-R and both VdTRP-R and AmTRP-R, respectively. A total of sixteen other sequences were included in this analysis, in which the *Drosophila* RYamide receptor served as the outgroup. The tree was inferred in MEGA 5, applying the Neighbor-Joining method with 1,000 bootstrap tests. The percentage of the 1000 bootstrap replicates supporting each node is indicated. The cDNA and translations for VdNTL-R, VdTRP-R and AmTRP-R are in Supplementary Data 1 to 3.

The open reading frames of the *V. destructor* NTL receptor (VdNTL-R), TRP receptor (VdTRP-R) and *A. mellifera* TRP receptor (AmTRP-R) consist of 461, 437 and 421 amino acids, respectively (Supplementary Info 1 to 3 and GenBank Accession Numbers: KT232310 to KT232312). A phylogenetic tree was constructed using the Neighbor-Joining method for the sequences of NTL-R and TRP-R available in GenBank, representing several insect species and mites (Fig. 3). The tree was clearly divided into 2 groups: one containing NTL-R and the other TRP-R, as was shown in a previous study^10^. VdTRP-R and AmTRP-R were grouped together with other TRP-R orthologs. As expected, VdTRP-R showed the closest relationship with the TRP-R of *M. occidentalis* and VdNTL-R shared the highest similarity with *M. occidentalis* NTL-R. The honey bee genome lacked both the NTL peptide and its receptor, implying true loss of this signaling system in the honey bee.

A noticeable unusual evolutionary pattern of the TRP/NTL signaling systems is the loss (or rapid divergent evolution) and gain of the ligands and receptors^10^. In Hymenoptera, *A. mellifera* and *Nasonia vitripennis* genomes lack both NTL and NTL-R, two *Bombus* species and *Megachile rotundata* genomes contain NTL-R, but not NTL (ref. 5 and therein for GenBank Accession numbers). Although the missing genes may be partly due to an incomplete sequence or to limitation in the search algorithm, major gene losses in NTL signaling in Hymenoptera have likely occurred. In contrast, an Acari: Acariformes, *Tetranicus urticae*, that is distantly related to the varroa mite, contains two genes encoding NTL/TRP precursors, each encoding the predicted mature peptides in a mixed array for both NTL and TRP^10^; *trp1* encodes two NTL-motif and one TRP-motif peptides, while *trp2* encodes one NTL-motif and one TRP-motif peptides. The *T. urticae* genome also contains four putative NTL-Rs likely by recent genome expansions^10^. The genome sequences of other species in the Acari: Parasitiformes, *Ixodes scapularis* and *M. occidentalis*, that are closer to the varroa mite, contain one-to-one orthologs for each NTL and TRP; IscW_ISCW021632 and XP_003739014 for NTL, and IscW_ISCW008383 and XP_003748097 for TRP.

### Functional Characterization of the Receptors

**Figure 4:**
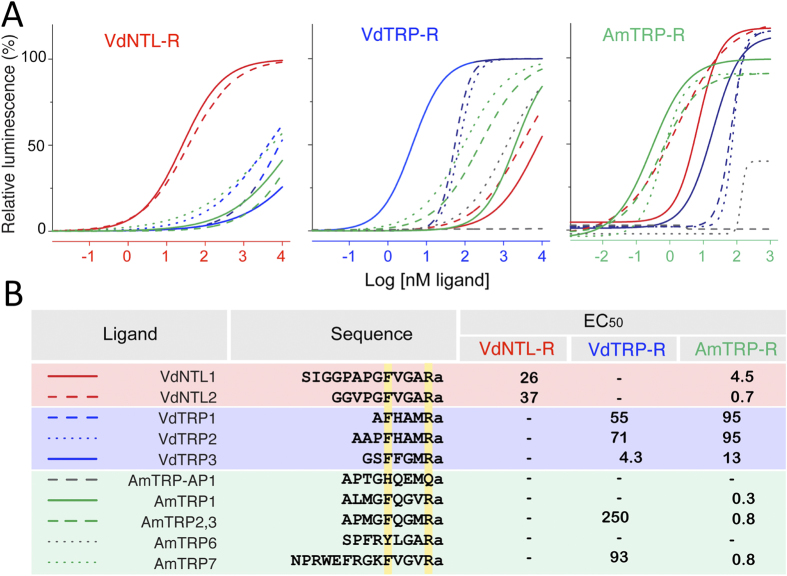
The receptor activities in response to authentic ligands confirmed the ligand-specific activities of the receptors with some degree of cross-reactivity. (**A**) Dose-response curves for three receptors, VdNTL-R, VdTRP-R and AmTRP-R, to 10 endogenous ligands. Red, blue, and green lines represent the activities of the VdNTL, VdTRP, and AmTRP mature peptides, respectively. Grey represents TRP-associated peptide (AmTRP-AP1). (**B**) Ligand names, sequences, and EC_50_s values deduced from the dose-response curves are shown. The table showing the summary of raw data is in Supplementary data 4.

Transient expression of the three receptors was successfully carried out in CHO-K1 cells carrying Ga16 and the reporter aequorin. In this system, ligand-mediated G-protein-coupled receptor (GPCR) activation initiates calcium mobilization that is indicated by the increased luminescence of aequorin. The endogenous ligands from each species clearly discriminated their respective receptors with high potencies (Fig. 4 and Supplementary Info 4); however, in general, the receptors from *V. destructor,* VdTRP-R and VdNTL-R, showed lower activities than AmTRP-R, implicating lower efficiencies in downstream coupling to the reporter system in VdTRP-R and VdNTL-R than in AmTRP-R in the CHO-K1 cells. For example, the EC_50_ of VdNTL-R was 26 nM for the putative authentic ligand VdNTL1, while that of AmTRP-R was at 0.3 nM of AmTRP2 (Fig. 4). Disregarding the potential bias caused by the intracellular coupling efficiencies in the heterologously expressed GPCR, the rank activity of the ligands on each receptor clearly indicates that the annotation of the receptor based on sequence similarity was correct.

VdNTL-R was highly specific to VdNTL peptides with more than a 100× difference versus the EC_50_s of other peptides. VdTRP-R was most sensitive to VdTRP3 (GSFFGMRa) and showed a >10× lower sensitivity to other VdTRPs and AmTRP7, and further lower sensitivities to VdNTLs. AmTRP-R exhibited the most promiscuous reactivities to other peptides. Specifically, VdNTL2 was equally potent as the AmTRPs (AmTRP1, 2, 3, and 7) on the AmTRP-R. Comparing VdNTL1 and VdNTL2 on the AmTRP-R, the approximately 6× differences in the activity must be determined by the N-terminal region of the amino acid residues because the C-terminal 7 amino acid residues are identical between the two peptides (PGFVGARamide). The promiscuity of AmTRP-R may be a consequence of the evolution of AmTRP and the receptor pair in the loss of the NTL system in this species. Therefore, AmTRP-R specificity for discriminating against NTL was not essential in the absence of the NTL system in its evolution.

**Figure 5:**
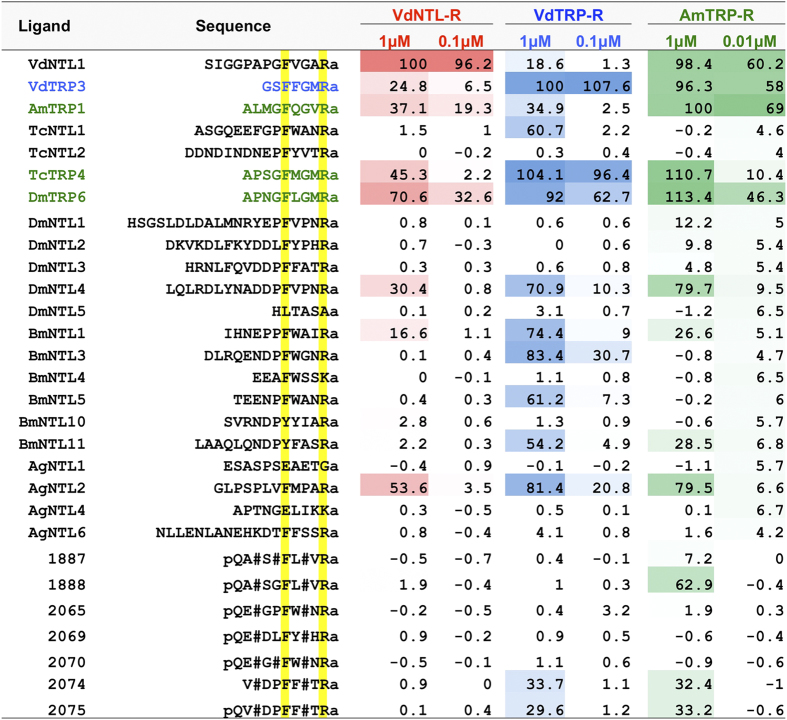
Agonistic activities of NTL and TRP peptides of various other insect species and peptidomimetics on the three receptors. All the ligands were tested at 2 concentrations. The data are relative luminescence unit (RLU) from 3 biological replications which were normalized by highest activity of the endogenous ligand for each receptor. The sequence is shown with the conserved sequences highlighted in yellow, other symbols show the modification of the peptidomimetics. pQ, pyroglutamate; #, Aib; a, amide.

In an expanded test of various ligands from other species and peptidomimetics, there were no ligands that specifically activated only the VdNTL-R, which was an original intention of this study. The assay provided robust results with the standard deviation within 20% in three biological replications. Mimetic analogs of TRPs, such as Leuma-TRP-1 (pQA[Aib]SGFL[Aib]VRamide, compound 1888 in Fig. 5), that feature multiple Aib (alpha-aminoisobutyric acid) residues have been shown to demonstrate markedly enhanced biostability and highly potent activity in a cockroach TRP myotropic bioassay, as well as potent oral aphicidal activity^18^. Given the close relationship between the TRP and NTL neuropeptidergic systems, a small set of NTL analogs were designed and synthesized that contain Aib residues in analogous positions. Leuma-TRP-1 proved selective by showing some activity on AmTRP-R, and failing to show agonistic activity on either VdTRP-R or VdNTL-R. NTL Aib analogs 2074 and 2075 (similar to DmNTL4^10^) showed low activity on both AmTRP-R and VdTRP-R, but not on the NTL receptor of the Varroa mite. Indeed, none of peptidomimetics tested in this study activated VdNTL-R. This failure may be due to the limited set of peptides and peptidomimetics that were synthesized and tested in this study. To find VdNTL-R-specific agonists, further expansion of the varied ligands based on the VdNTL sequence, including N-terminal variants, is needed. In addition, testing of the Aib-containing peptidomimetics for antagonistic activity on the GPCRs may provide additional information for development of compounds with acaricidal potential.

Surprisingly, we found a number of ligands that specifically activated only VdTRP-R: TcNTL1 (ASGQEEFGPFWANRa), BmNTL3 (DLRQENDPFWGNRamide) and BmNTL5 (TEENPFWANRamide). The consensus C-terminal sequence of these peptides PFW(A,G)NRamide is likely the determinant of the specificity. A previous study describing taxon-specific NTL motifs showed that W (Trp) in the X1 position is common in Lepidoptera, Coleoptera, and Hemiptera. N (Asn) in the X3 position was also frequently found in Diptera, Lepidoptera, and Coleoptera.

In conclusion, we identified peptide ligands that are specific to VdTRP-R, while they have no activities on AmTRP-R. We believe that the sequence motif (-PFW(A,G)NRamide) can be further developed for efficient peptidomimetics for a honey-bee-safe acaricide. Although we were unable to identify the specific agonist for VdNTL-R in our tests of a limited set of peptides and peptidomimetics, expanded searches with N-terminally varied peptides will help determine the ligand with enhanced selectivity. In addition, future work includes investigations of whether the peptidomimetics have antagonistic acitivities and whether those show toxic activities at the organismal level.

## Methods

### Chemicals and Insects

The peptides from *V. destructor* and *A. mellifera* were synthesized by Genescript (Piscataway, NJ) for higher than 75% purity. All the peptide mimetics were designed, synthesized, characterized and purified by Nachman and coworkers according to previously reported procedures^18^. To culture Chinese hamster ovary (CHO) cells, DMEM/F12 medium, fetal bovine serum (FBS), Fungizone^®^ and Penicillin/Streptomycin, and coelenterazine for an aequorin functional assay were purchased from Gibco^®^ Cell Culture at Life Technologies^TM^ (Grand Island, NY). TransIT^®^-LT1 Transfection Reagent (Mirus Bio LLC, Madison, WI) was used for the transient transfections. Approximately 100 adult mites of the *V. destructor* were collected from the bee hives in Manhattan, Kansas. The mites were dissected in PBS to obtain the synganglions and salivary glands for the immunohistochemistry of NTL and tachykinin.

### Identification of NTL, TRP and their Receptors

In a BlastP search using the *Drosophila* data against the nr database in GenBank, we found NTL-like, tachykinin-like precursors as well as their receptors in the western predatory mite (*M. occidentalis*) genome. The sequences of NTL, TRP and their receptors obtained from the GenBank were used as the query sequences for the searches of the *V. destructor* genome data that is available in BeeBase (http://hymenopteragenome.org/beebase/, Amel_4.5)^20^. Manual annotations of the genes encoding NTL, TRP and their receptors in *V. destructor* were made on the sequences of the corresponding scaffolds. Similarly, the *Apis* TRP and receptor were obtained from the genome data available in GenBank on the NCBI website, using *Drosophila* sequences as the query sequence.

### Molecular Cloning and Sequence Analysis

Honey bee workers for total RNA isolation were collected from the Insect Zoo at Kansas State University. Varroa mites were collected from beehives in Manhattan, Kansas. Total RNA was extracted using Tri Reagent (Molecular Research Center Inc.) with a treatment with DNaseI (Ambion) followed by a phenol-chloroform (Fisher Scientific) extraction. Approximately 1 μg total RNA was used for cDNA synthesis. The first strand cDNA was synthesized using the ImProm-II™ Reverse Transcription System for RT-PCR with random hexamers in a total volume of 20 μL, according to the manufacturer’s instructions (Promega). The 1^st^ strand cDNA was used as template to amplify the target receptors using a high-fidelity DNA Polymerase, PrimeSTAR^TM^ HS (Takara). Primers for nested PCR of each receptor amplification were designed based on the 5′ and 3′ ends of the open reading frames of the candidate target genes (Table 1). The total reaction volume of 50 μL included approximately 50 ng of cDNA, 10 μL of 5× PrimeSTAR buffer with Mg^2+^, 0.32 mM of each dNTP, and 0.2 μM of each primer. The PCR program included 35 cycles: 98 °C for 10 sec, 56 °C for 10 sec and 72 °C for 90 sec with a final extension of 6 min at 72 °C. PCR products were purified using the Zymo PCR clean up kit (Zymo research) and subcloned into the pGEMT easy vector (Promega). For sequence comparisons, sequence alignments were made by ClustalW2 (http://www.ebi.ac.uk/Tools/msa/clustalw2/), and the phylogenetic tree was constructed by MEGA5^21^ applying the Neighbor-Joining method with a bootstrap test of 1000 replications and complete deletion of the gaps in the aligned sequence.

To test for tissue-specific expression patterns of *VdNTL, VdTR,* and *VdRPS3* primers designed across exon-exon junctions were used to detect the expression levels of *VdNTL* and *VdTRP* in the synganglion and salivary gland. The PCR procedure was performed as described above. The results from quantitative PCR using SYBR Green-method were analyzed by delta-delta CT methods for three technical replications. The sizes of amplicons were confirmed on an agarose gel.

### Heterologous Expression, GPCR and Functional Assays

The PCR amplicons of the full open reading frames (ORFs) of the three receptors were initially cloned into the pGEMT easy vector (Promega) and transferred to the expression vector pcDNA3.1(+) (Invitrogen) using the common restriction enzymes in the multi-cloning site (*EcoR*I for AmTRPR, *Not*I for both VdNTLR and VdTRPR). The sequences of the inserts were confirmed by Sanger sequencing prior to heterologous expression. High-quality plasmid DNA prepared using the plasmid MIDIprep kit (Qiagen) was used for transient transfection. The methods for transient expression of aequorin and G-alpha16 in Chinese hamster ovary (CHO-K1) cells and the procedures for the assays were previously described^10^,^22^,^23^,^24^. Thirty hours after the transfection, the cells were collected and preincubated with the coelenterazine (Invitrogen) for the functional assay as previously described^10^,^22^. Serial dilutions (10-fold, ranging from 0.001 to 1000 nM) of the 10 endogenous ligands were used for treatment of the cells. These ten natural ligands included two VdNTL peptides and three VdNTL peptides predicted from the respective putative precursors and also included five AmTRP peptides. The elevated luminescence caused by intracellular calcium mobilization was measured for a continuous 20 seconds at every 100-ms interval. Dose response curves and EC_50_s of each ligand for each receptor were obtained by logistic fitting in Origin 8.6 (OriginLab).

In the test of an expanded set of ligands including peptidomimetics, we measured the relative activities of the ligands on each receptor that were normalized by the activities of the endogenous ligand with the highest activity (10 μM of each); i.e., VdNTL1 on VdNTL-R, VdTRP3 on VdTRPR, and AmTRP1 on AmTRP-R (Fig. 5). The test ligands were mainly the NTL peptides of *T. castaneum, D. melanogaster, B. mori*, and *Anopheles gambiae* and two TRPs, TcTRP4 and DmTRP6. Two doses of each ligand were tested for each receptor: 1 and 0.1 μM for VdNTL-R and VdTRP-R and 1 and 0.01 μM for AmTRP-R.

### Immunohistochemistry

For immunohistochemistry of the synganglion of the varroa mite, three antibodies were used: rabbit anti-DmNTL4, mouse anti-DmNTL5, and rabbit anti-LmTRP1 (a gift from Dr. Liliane Schoofs, KU Leuven, Belgium). Further details for each antibody are available in the original publications^10^,^19^. The procedure is modified from the method established for the tick synganglion^25^,^26^,^27^.

The adult mites were dissected in ice-cold phosphate-buffered saline (PBS: 137 mM NaCl, 1.45 mM NaH_2_PO_4_, 20.5 mM Na_2_HPO_4_, pH 7.2). The dissected synganglion was fixed in Bouin’s solution (37% formaldehyde and a saturated solution of picric acid in a ratio of 1:3) at 4 °C overnight. The fixed samples were washed in PBS containing 0.5% Triton X-100 (PBST). The tissues were then preadsorbed with 5% normal goat serum (Sigma) in PBST for 10 minutes and subsequently incubated with anti-DmNTL4 (1:1000), anti-DmNTL5 (1:1000) or anti-LomTK1 (1:500) antibodies for 2 days at 4 °C. After three washes with PBST (5 min each), the tissues were incubated overnight in the goat anti–rabbit or goat anti-mouse secondary antibody (conjugated with Alexa Fluor 488, Molecular Probes). The tissues were washed in PBST and finally mounted on a slide glass in glycerol. The images were captured on a confocal microscope (Zeiss LSM 710). Schematic drawings were made in Adobe Photoshop 7.0 or Canvas 8.0. The data presented show the staining patterns commonly found in multiple samples in three trials of more than 10 individuals each.

## Additional Information

**How to cite this article**: Jiang, H. *et al.* Ligand selectivity in tachykinin and natalisin neuropeptidergic systems of the honey bee parasitic mite *Varroa destructor. Sci. Rep.* **6**, 19547; doi: 10.1038/srep19547 (2016).

## Supplementary Material

Supplementary Information

## Figures and Tables

**Table 1:** Primers used in this study.

**Experiments**	**Genes**	**GenBank Accession Numbers**	**Primers**	
Cloning			1^st^ round PCR	Nested PCR
	AmTRPR	KT232312	F1: GTGTGGAAAGTGATACGTTC	F2: GATGCAGACCGTAGAAGTT
			R1: CAAGTATGTACTCGTTGCTG	R2: GTCAAGACACGTGACCCG
	VdNTL-R	KT232310	F1: CAGGTTTCCACAGAGCGTC	F2: CACAAGGGCCAAGGTTAAC
			R1: CTGTTCCACTTTAGCCTACC	R2: GCTTTATTTGCCTAGAGCAC
	VdTRP-R	KT232311	F1: GAGCTTATTAAACTGGCTCG	F2: GCCTCATAATGGATGTCCTC
			R1: GCCACGAACTAATCAGAAAC	R2: GCTACAGTGTAGTCTAACCCTTC
Tissue-specific RT-PCR	VdNTL		F: ATACCTTTACGATGATTTGCTG	R: CTCCGCCCTTTTTACCAC
	VdTRP		F: AGGCAAGAAAGCCGCACC	R: CGCATTCCGAAGAACGAT
	VdRPS3		F: GGGCGTAGAGGTGCGCAACGG	R: CGACACCTTCTCGGCGAACAG

For receptor amplifications, two pairs of primers for nested PCR are shown.
