# Supplementary material for: Ligand selectivity in tachykinin and natalisin neuropeptidergic systems of the honey bee parasitic mite Varroa destructor
Source: Sci Rep. 2016 Jan 28;6:19547. doi: 10.1038/srep19547 (PMC4730192; doi:10.1038/srep19547)
Supplement: Supplementary Information [file srep19547-s1.pdf]

Supplementary Data

Ligand selectivity in tachykinin and natalisin neuropeptidergic systems of the honey bee parasitic mite *Varroa destructor*

Hongbo Jiang[a, b], Donghun Kim[a], Sharaon Dobesh[a], Jay D. Evans[c], Ronald J. Nachman[d], Krzysztof Kaczmarek[d,e], Janusz Zabrocki[d,e], and Yoonseong Park[a*]

[a] *Department of Entomology, Kansas State University, Manhattan, Kansas 66506, United States*

[b] *Key Laboratory of Entomology and Pest Control Engineering, College of Plant Protection, Southwest University, Chongqing 400715, People's Republic of China*

[c] *Bee Research Laboratory, BARC-E, USDA-Agricultural Research Service, Beltsville, MD 20705, USA*

[d] *Insect Control and Cotton Disease Research Unit, Southern Plains Agricultural Research Center, USDA, 2881 F/B Road, College Station, TX 77845, United States*

[e] *Institute of Organic Chemistry, Lodz University of Technology, 90-924 Lodz, Poland*

Supplementary Data 1

The cDNA and deduced amino acid sequences for tachykinin receptor of *Apis melifera* (AmTRP-R).

1	ATGCAGACCGTAGAAGTTTTTCTAAACTCCACCGTCTCCGACCCGTGGAACGTCTCTTTATTGATGGAGAATTAC
1	M Q T V E V F L N S T V S D P W N V S L L M E N Y
76	ACTTACCACAATAACACGAATATCACGGATTTGCAATTAAGAAATCAATTCATACTTCCGTGGTGGAGGCAAATG
26	T Y H N N T N I T D L Q L R N Q F I L P W W R Q M
151	ATATGGACGTTATTGTTCGCCGGTATGATCATAGTTGCAACCGGTGGTAATCTGATAGTGATTTGGATAGTGATG
51	I W T L L F A G M I I V A T G G N L I V I W I V M
226	GCCCACAAACGGATGCGTACAGTCACTAATTACTTCTTGGTAAACTTGAGCATCGCGGACGCCATGGTGTCCACG
76	A H K R M R T V T N Y F L V N L S I A D A M V S T
301	TTGAACGTCACGTTCAATTACGTCTACATGTTGAACAGCCATTGGCCTTTTGGCACCCTTTACTGCAAGATCTGC
101	L N V T F N Y V Y M L N S H W P F G T L Y C K I C
376	CAGTTCGTCGCTGTCCTCACCATATGCGCCAGCGTCTTCACTTTAATGGCGATTTCCATCGACAGATACATGGCC
126	Q F V A V L T I C A S V F T L M A I S I D R Y M A
451	ATAATGAACCCTTTGAGGCCGCGCATGGGCAAAAGGGCGACGATTTGCATAGCCATCGTTATTTGGATCGTGGGG
151	I M N P L R P R M G K R A T I C I A I V I W I V G
526	GCCGTGTTGTCGCTGCCGATGCTACTCTTCTACAGGACTTACACCCAGAATTTCGTGAATGGAGAAGTACGAGTT
176	A V L S L P M L L F Y R T Y T Q N F V N G E V R V
601	ATATGCTACGGCGATTTCCCGAACAGGGATGACAATGGCCTCAGTTACGACGAATATTTGTACAACGTGATCTTC
201	I C Y G D F P N R D D N G L S Y D E Y L Y N V I F
676	ATGGTGTTGACGTACGTTCTACCAATTGGATCGATGACATTCACGTACGCAAGGATCGGTTTGGAGCTGTGGGGC
226	M V L T Y V L P I G S M T F T Y A R I G L E L W G
751	TCGCAAAGCATCGGCGAAAATACCGCGGGCCAATTGGAGGGTATAAGGAGCAAACGAAGGGTGGTAAAAATGATG
251	S Q S I G E N T A G Q L E G I R S K R R V V K M M
826	ATCGTGGTGGTATTAATATTTGCTATATGCTGGTTACCTTTTCACGTGTACTTCATCATAACGTCGTATTTTCCG
276	I V V V L I F A I C W L P F H V Y F I I T S Y F P
901	GAGGTTACGAACGAATCGTACATCCAAGAAGTTTTCTTGGGCATTTACTGGCTTGCGATGTCTAACAGCATGTAT
301	E V T N E S Y I Q E V F L G I Y W L A M S N S M Y
976	AATCCCATAATCTACTGTTGGATGAACTCCAGATTTCGACGCGGATTCGCCCATTTTTTCTCGTGGTGCCCTATG
326	N P I I Y C W M N S R F R R G F A H F F S W C P M
1051	GTGAAAGTTCCGCCCGAGCCCTCGTTATCACGATCGGAGGCGTTAACGTCGCGATACAGTTGCACAGGAAGTCCC
351	V K V P P E P S L S R S E A L T S R Y S C T G S P
1126	CAGACCAACACAAGGATATCACGCAACGGTACACCACGAATACATATGTGTCTACGATCGAGAGGGGGAAACGAT
376	Q T N T R I S R N G T P R I H M C L R S R G G N D
1201	CGATTTTTGGGCGAGCATCGAGGAAGGAAGTGGAAAACCTCGCAAACTACGGGTCACGTGTCTTGA
401	R F L G E H R G R K W K T S Q T T G H V S *

Supplementary Data 2

The cDNA and deduced amino acid sequences for tachykinin receptor of *Varroa descructor* (VdTRP-R).

```
1       ATGGATGTCCTCGAGGAGATGAATCTAACCTTTTTTAATATATCGGAGGTGTTCAAAATATATAGATCTAACTTC
1        M  D  V  L  E  E  M  N  L  T  F  F  N  I  S  E  V  F  K  I  Y  R  S  N  F

76      TCCTTCGAGGATACCGACTATGCGCTCTTCATGCCCATCTATATGGAGGTGATATGGTGCATCCTGTTTTCCGTG
26       S  F  E  D  T  D  Y  A  L  F  M  P  I  Y  M  E  V  I  W  C  I  L  F  S  V

151     ATGATCGTCGTTGCAGCCTGTGGTAATCTCATCGTAATTTGGATTGTCTTAGCTCACAAACGAATGCGTACCGTA
51       M  I  V  V  A  A  C  G  N  L  I  V  I  W  I  V  L  A  H  K  R  M  R  T  V

226     ACCAATTACTTCATCGTGAATCTGTCAATAGCCGATACGATGGTGTCAACGCTCAACGTTATTTTTAACTTTACG
76       T  N  Y  F  I  V  N  L  S  I  A  D  T  M  V  S  T  L  N  V  I  F  N  F  T

301     TTTATGCTACGGAGCGAATGGTGGTTCGGGGAATGGTACTGCAAATTTAGTAACTTTGTCGCTCTCGTCAGTGTA
101      F  M  L  R  S  E  W  W  F  G  E  W  Y  C  K  F  S  N  F  V  A  L  V  S  V

376     TCGGCAAGTGTGTTTACTCTAATGGCTATCTCAATTGATCGGTATATGGCCATCATGCATCCCCTTCACCCTCGA
126      S  A  S  V  F  T  L  M  A  I  S  I  D  R  Y  M  A  I  M  H  P  L  H  P  R

451     ATGTCCCGTATGATGACACTCAATATCGCGCTGTGCATTTGGCTGTTAGCGGGTTTGCTCGCATGTCCGCAGTAC
151      M  S  R  M  M  T  L  N  I  A  L  C  I  W  L  L  A  G  L  L  A  C  P  Q  Y

526     GTCTATTCAAGAGTTAAAGAGCAAGACAACCATACCGTTTGCTACATGTTCCTTGATGAAGATGGAGAAATTACC
176      V  Y  S  R  V  K  E  Q  D  N  H  T  V  C  Y  M  F  L  D  E  D  G  E  I  T

601     GAAAGTCGCGCCGATTACCTATACAACCTCGTGGTCCTGATAGTAACTTATATCATCCCTATGCAGGCTATGGCT
201      E  S  R  A  D  Y  L  Y  N  L  V  V  L  I  V  T  Y  I  I  P  M  Q  A  M  A

676     TTTACGTATTTTCGTGTTGGCCGTGAGCTTTGGGGACAGCAAAGCATCGGTGAAGTCACTCGAAAACAAACCGAG
226      F  T  Y  F  R  V  G  R  E  L  W  G  Q  Q  S  I  G  E  V  T  R  K  Q  T  E

751     GCAATTAATTCTAAACGAAAGATCGTTAAAATGATGATTGTGATTGTCGCCATTTTCGGTGTGTGTTGGCTGCCC
251      A  I  N  S  K  R  K  I  V  K  M  M  I  V  I  V  A  I  F  G  V  C  W  L  P

826     TATCATCTCTACTTTTTGCTGGTGCATCATTACCCTGACATGCGCAACTCTGTCTACATCCAAAATATCTACTTA
276      Y  H  L  Y  F  L  L  V  H  H  Y  P  D  M  R  N  S  V  Y  I  Q  N  I  Y  L

901     ACCATCTACTTTCTAGCCATGTCGAATTCGATGTACAACCCTGTCATTTACTGCTGGATGAACAGCAGGTTCCGC
301      T  I  Y  F  L  A  M  S  N  S  M  Y  N  P  V  I  Y  C  W  M  N  S  R  F  R

976     GAGGGCTTTAAAGCGGTGTTCTGCTGCTATGCATTGACTGGCAAACAATTGAGCGCATTCGCCAAGAACAACCGG
326      E  G  F  K  A  V  F  C  C  Y  A  L  T  G  K  Q  L  S  A  F  A  K  N  N  R

1051    AAGTTTGCGCGGTATTCGTGTGCGTCCGAACCATATTCAACCACCCGTGTCACGCTAAATCATCTACACACCACC
351      K  F  A  R  Y  S  C  A  S  E  P  Y  S  T  T  R  V  T  L  N  H  L  H  T  T

1126    CAAAACATTAACAATCATCAGCTAATCTCGAATCAAGCCGACATCGATCTGCAGCATCAGCCCGATGGGTCAGCT
376      Q  N  I  N  N  H  Q  L  I  S  N  Q  A  D  I  D  L  Q  H  Q  P  D  G  S  A

1201    TCTACAGAACCTGGAACTGGTGAAAACGGTATTCACCGACTCTTGCGAAGCCAACAAATCCGAATACAGCGCAAC
401      S  T  E  P  G  T  G  E  N  G  I  H  R  L  L  R  S  Q  Q  I  R  I  Q  R  N

1276    AGCCAGTTACCCTTTGCTAACGGACACACTGAAGTATAA
426      S  Q  L  P  F  A  N  G  H  T  E  V  *
```

Supplementary Data 3

The cDNA and deduced amino acid sequences for natalisin receptor of *Varroa descructor* (VdNTL-R).

```
1       ATGACCTTCACGATGAATCAGCTGGCAGCCGTCGCCCTCAACACGACGGTGAGCCGGTGTTTGAAAGCCCTCAAT
1        M  T  F  T  M  N  Q  L  A  A  V  A  L  N  T  T  V  S  R  C  L  K  A  L  N

76      GATTCGCTATCAACAACGTCACCTGCCGCCGAAGTACCAGCCGATTGCGTATCGTTGTTTGACAACGTCACCAGC
26       D  S  L  S  T  T  S  P  A  A  E  V  P  A  D  C  V  S  L  F  D  N  V  T  S

151     GCTGATGGCAACATCACGGACTTGACAAGTCTTGGCAACTCAAGCTTTCCCGGCTATGTTGAGGGTGGATCAAGT
51       A  D  G  N  I  T  D  L  T  S  L  G  N  S  S  F  P  G  Y  V  E  G  G  S  S

226     CTGACATTTCTTCTGCCAATGTGGCAGCAGGTAACGTTCATCATGTGCTTTTCATTAATAGTGTTCGCAGGAATA
76       L  T  F  L  L  P  M  W  Q  Q  V  T  F  I  M  C  F  S  L  I  V  F  A  G  I

301     CTCGGCAATGCGATCGTTATCTGGATTGTACTCGCCCATCAGAGAATGCGAACGGTGACAAACTATTTTCTAGTG
101      L  G  N  A  I  V  I  W  I  V  L  A  H  Q  R  M  R  T  V  T  N  Y  F  L  V

376     AATCTATCAGTGGCTGACCTGACGACGGCCATGTTCAATGTGATTTTTAACGCCGTGTTTATGATGCATTCGCAT
126      N  L  S  V  A  D  L  T  T  A  M  F  N  V  I  F  N  A  V  F  M  M  H  S  H

451     TGGCCGTTCGGCGCATTGTACTGTCGAATTACCAATTTTATTTCGTTATTAACAGTGACCTCGTCCGTCTTCACA
151      W  P  F  G  A  L  Y  C  R  I  T  N  F  I  S  L  L  T  V  T  S  S  V  F  T

526     ATTAAGGCGATGAGTATTGATAGATGCATCGCGATTTACAACCCACTATCGCATCGGTTATCACGTAGATGTGCT
176      I  K  A  M  S  I  D  R  C  I  A  I  Y  N  P  L  S  H  R  L  S  R  R  C  A

601     CTAATTATTGTAGCATTAATATGGCTTGTTAGTTGCGTCATTGCGCTACCAGGGTATGCGTTCGCCCAGACGCGA
201      L  I  I  V  A  L  I  W  L  V  S  C  V  I  A  L  P  G  Y  A  F  A  Q  T  R

676     AGTTATGAAGATCGCATCGTTTGTTCACTGAGTTGGCCGGAGCTCGGCGGAGTCAATCCGAGTCAAGTTGACTTC
226      S  Y  E  D  R  I  V  C  S  L  S  W  P  E  L  G  G  V  N  P  S  Q  V  D  F

751     ATTTACAATGTCGTGTTTATGATCGCCACGTATTTTGTGCCAATGGTCACGATTGCTATTGCCTATTCAATTATG
251      I  Y  N  V  V  F  M  I  A  T  Y  F  V  P  M  V  T  I  A  I  A  Y  S  I  M

826     GGCCACGTGCTCTGGCGGAGCAAAGGTATCGGGGAGCAGACCGAGCGGCAGAAGGAGGCCATACGATCCAAACAA
276      G  H  V  L  W  R  S  K  G  I  G  E  Q  T  E  R  Q  K  E  A  I  R  S  K  Q

901     CGGGTGGTCCGTATGCTTGTTGTCGTTGTGATGATCTTCGGAGTGTGCTGGCTCCCTTACCATCTTTACTTCATC
301      R  V  V  R  M  L  V  V  V  V  M  I  F  G  V  C  W  L  P  Y  H  L  Y  F  I

976     TACACGTACCTCGATCCGGATGTGACGTACACAACATGGGCGCAGCCCTTATACCTAGTCATCTATTGGCTGGCC
326      Y  T  Y  L  D  P  D  V  T  Y  T  T  W  A  Q  P  L  Y  L  V  I  Y  W  L  A

1051    ATGTCCAACTGCATGTATAATCCATTTATCTACTACTGGATGAATTCTAGATTTCGTGGATATTTCCGTTACGTG
351      M  S  N  C  M  Y  N  P  F  I  Y  Y  W  M  N  S  R  F  R  G  Y  F  R  Y  V

1126    TTATGCTATTGCTGCAATCTTGGTAGTGGACTTGGCCACAAGTGCAGTGAAGTTGATCGACTTCAGAGCGACTGG
376      L  C  Y  C  C  N  L  G  S  G  L  G  H  K  C  S  E  V  D  R  L  Q  S  D  W

1201    ACGTTCAACAATAATTCGCCGCATCGACGCACTCTGAAAAGTACGGCCGGAACCGAGTGTCTCGTCCTTACGACT
401      T  F  N  N  N  S  P  H  R  R  T  L  K  S  T  A  G  T  E  C  L  V  L  T  T

1276    CCACCGAACAATCGGAATGCCACCGGGAGCCAGCAAAAACACCCGCACAGCCACAAGAACGGCAGCTGGCAGGTA
426      P  P  N  N  R  N  A  T  G  S  Q  Q  K  H  P  H  S  H  K  N  G  S  W  Q  V

1351    CATAGTGACTCTTCAACAGTTACCTATGTGCTCTAG
451      H  S  D  S  S  T  V  T  Y  V  L  *
```

Supplementary Data 4

Receptor activities measured by the luminescence in response to endogenous ligands. Activities of three receptors, VdNTL-R, VdTRP-R and AmTRP-R, to 10 endogenous ligands at each dose are shown. The dose-response curves and EC50 are found in Figure 4 of the main text.

VdNTL-R

	Dose (log[nM ligand])											
	-1		0		1		2		3		4	
	Avg	SD	Avg	SD	Avg	SD	Avg	SD	Avg	SD	Avg	SD
VdNTL1	6.05	2.02	5.34	1.78	32.88	5.48	72.46	8.05	100.00	11.76	107.41	12.64
VdNTL2	2.27	0.76	10.09	3.36	29.47	4.91	62.26	6.92	97.41	11.46	106.12	12.48
VdTRP1	9.94	3.31	2.14	0.71	4.59	0.77	0.57	0.06	17.88	2.10	52.80	6.21
VdTRP2	6.53	2.18	3.02	1.01	5.36	0.89	8.13	0.90	32.51	3.82	61.25	7.21
VdTRP3	-0.15	0.05	4.70	1.57	6.65	1.11	3.52	0.39	6.95	0.82	26.63	3.13
AmTRP-AP1	1.64	0.55	1.61	0.54	-0.64	0.11	-4.24	0.47	-2.67	0.31	-1.19	0.14
AmTRP1	-1.39	0.46	-1.16	0.39	-1.03	0.17	2.27	0.25	20.67	2.43	39.86	4.69
AMTRP3	-2.49	0.83	-2.80	0.93	-3.51	0.58	0.78	0.09	10.87	1.28	32.98	3.88
AmTRP6	-2.27	0.76	-4.95	1.65	-2.94	0.49	-1.87	0.21	-0.24	0.03	1.44	0.17
AmTRP7	-2.82	0.94	-1.57	0.52	-3.04	0.51	17.12	1.90	40.55	4.77	51.60	6.07

VdTRP-R

	Dose (log[nM ligand])											
	-1		0		1		2		3		4	
	Avg	SD	Avg	SD	Avg	SD	Avg	SD	Avg	SD	Avg	SD
VdNTL1	1.07	0.99	0.36	0.21	-1.27	5.46	1.26	5.06	17.82	6.79	54.49	10.28
VdNTL2	0.62	0.65	1.13	0.30	0.99	5.54	2.82	5.81	35.52	8.25	68.47	11.13
VdTRP1	1.61	0.01	0.89	0.47	2.04	5.66	79.01	5.35	95.32	5.69	99.09	7.66
VdTRP2	1.68	0.60	3.13	0.53	1.80	8.29	67.20	12.25	100.00	15.00	107.57	15.74
VdTRP3	6.12	0.23	17.02	1.01	70.11	7.95	100.00	11.23	95.97	14.74	106.19	15.61
AmTRP-AP1	0.22	0.16	2.47	0.16	0.43	5.06	1.16	5.42	1.07	5.27	1.07	5.12

AmTRP1	0.40	0.14	1.33	0.12	0.57	5.10	2.42	5.23	33.46	7.07	83.23	8.99
AMTRP3	0.18	0.25	2.04	0.28	1.19	5.35	40.81	5.08	66.63	6.09	101.60	8.30
AmTRP6	-0.45	0.23	-0.75	0.49	2.68	5.29	9.23	5.19	48.29	5.02	81.75	5.14
AmTRP7	1.29	0.28	2.95	0.16	11.23	5.30	55.07	6.71	85.60	9.06	85.99	10.16

AmTRP-R

	Dose (log[nM ligand])											
	-2		-1		0		1		2		3	
	Avg	SD	Avg	SD	Avg	SD	Avg	SD	Avg	SD	Avg	SD
VdNTL1	4.77	0.99	6.98	0.80	9.79	6.06	76.19	10.30	106.11	9.94	124.03	12.36
VdNTL2	0.45	0.88	19.30	0.14	56.04	6.46	79.48	6.41	122.70	8.81	112.54	9.14
VdTRP1	0.40	0.01	3.43	1.80	2.89	5.80	3.82	9.71	81.18	9.05	115.60	8.65
VdTRP2	2.11	2.01	3.28	7.53	0.88	9.38	2.14	10.78	77.12	9.84	114.95	11.37
VdTRP3	-1.01	6.11	4.30	9.00	4.64	9.75	39.52	11.07	96.27	10.97	111.67	11.92
AmTRP-AP1	1.68	0.39	4.46	0.19	3.70	0.33	-0.91	0.65	0.65	0.64	1.07	4.08
AmTRP1	-0.39	8.09	28.18	9.41	70.94	11.47	93.62	10.58	97.98	11.53	100.00	11.20
AMTRP3	1.54	7.49	17.13	10.23	57.43	9.62	77.37	10.83	98.30	10.72	86.01	12.57
AmTRP6	-1.94	0.44	-1.52	0.24	-3.39	0.27	-2.48	0.36	2.81	3.69	39.98	8.79
AmTRP7	-1.68	4.70	3.26	7.74	58.70	10.22	86.20	10.04	90.29	10.46	93.33	9.99
